# Unilateral congenital non-syndromic retinal vessel dilation and tortuosity

**DOI:** 10.1016/j.ajoc.2021.101160

**Published:** 2021-07-07

**Authors:** Ethan Waisberg, Michalis Georgiou, Michel Michaelides, Ranjan Rajendram

**Affiliations:** a UCL Institute of Ophthalmology, University College London, 11-43 Bath Street, London, EC1V 9EL, UK; b Moorfields Eye Hospital NHS Foundation Trust, City Road, London, EC1V 2PD, UK; c UCD School of Medicine, University College Dublin, Belfield, Dublin 4, Ireland

**Keywords:** Aberrant retinal vessels, Retinal vessel, Optical coherence tomography angiography, Vessel dilation, Arteriovenous malformation

## Abstract

**Purpose:**

To present a case of atypical unilateral developmental retinal vascular anomaly.

**Observations:**

A 10-year-old girl presented to her paediatrician after an absent red reflex was noted in a photograph. She had right anisometropic amblyopia and right iris heterochromia, but was otherwise healthy, with no visual complaints. Fundus examination revealed abnormal right retinal vasculature in keeping with an arteriovenous malformation (AVM). OCTA performed at age 16, showed large aberrant veins in the right eye, whereas OCTA B-Scans showed that the same eye had significantly higher retinal blood perfusion than the unaffected eye.

**Conclusions and Importance:**

OCTA is a valuable, non-invasive emerging method of evaluating patients with AVMs, with this patient having a unique unilateral presentation of a developmental anomaly, without evidence of progression or other vessel malformation. OCTA allowed assessment of flow between the affected and non-affected eye, quantifying the greater blood perfusion in the affected eye due to the AVM.

## 1 Introduction

A retinal arteriovenous malformation (AVM) refers to a congenital abnormality of the retinal vasculature with arteriovenous shunting and the capillary system being bypassed. This may result in significant reduction of visual acuity. Congenital retinal macrovessels (CRM) is a venous malformation of the retina that is associated with brain vessel abnormalities, which can present similarly to AVMs. However, usually diagnosis is based on a retinal vessel crossing the macula that passes both below and above the horizontal raphe and usually presents monocularly.^1^ The goal of this case report is to compare retinal blood perfusion between both eyes in a patient with AVM.

## 2 Case report

**Fig. 1:**
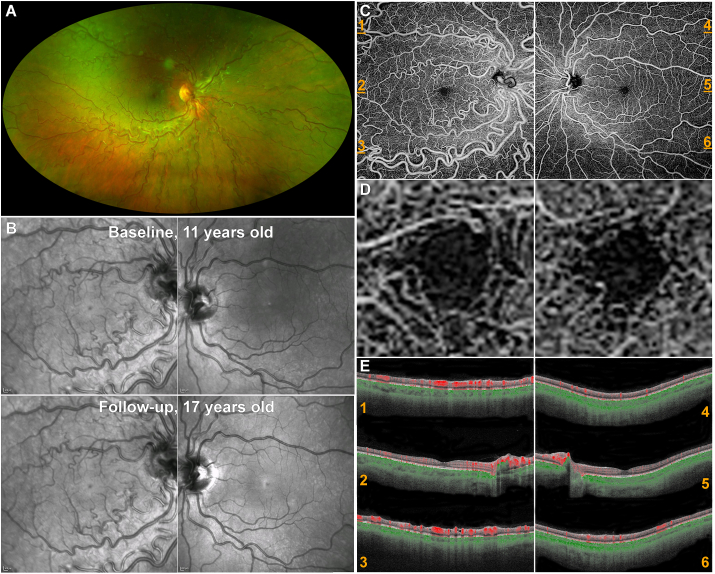
Multimodal Retinal Imaging of a case of unilateral vessel malformation. **(A)** Ultra-widefield (200°) confocal scanning laser color fundus imaging (Optos plc, Dunfermline, UK) of the right eye with evident vessel tortuosity and dilation at 11 years of age. **(B)** Infrared image (IRR Spectralis, Heidelberg Engineering Ltd, Heidelberg, Germany) of both eyes at 11 years old, and after 6 years of follow-up, without evidence of change. **(C)** Swept-source OCT Angiography (SS-OCTA, PLEX Elite, Zeiss) of the superficial capillary plexus for both eyes (12mm square scan, centered to the fovea). Turbulence in the vasculature causes variances in the images, which is depicted as vessels. The right eye has an arteriovenous malformation and displays microvascular capillary irregularities (such as large aberrant veins). An increased vascular blood flow was observed in the large aberrant vessels. The patient's vision in the left eye was normal and has no significant abnormalities. **(D)** Ten times magnification of the SS-OCTA images at **(C),** of the foveal avascular zone (FAZ). The aberrant vessels do not have an impact on the size or shape of the FAZ. **(E)** Horizontal optical coherence tomography angiography B-scans of the right and the left eye over the superior arcade (1,4), fovea (2,5) and inferior arcade (3,6). These numbered images correspond to the locations marked with orange dashes in **(C)**. The color-coded SS-OCT B-scans can clearly show blood flow in the superficial (red) and deep (green) capillary plexuses. The highly saturated clusters of red are veins. The right eye has significantly higher retinal blood perfusion than the unaffected eye. The percentage difference of blood flow between the left and right eye reached a maximum of 1102% in the superior arcade (1 and 4). (For interpretation of the references to colour in this figure legend, the reader is referred to the Web version of this article.)

A 10-year-old female with a history of iris heterochromia and anisometropic amblyopia (diagnosed at age 6) presented to her paediatrician after she had an absent red reflex noted in a photograph. The patient had no notable family medical history and an otherwise unremarkable personal medical history, including no history of cyanotic disease. The patient was referred to our tertiary eye centre for evaluation (Moorfields Eye Hospital, London, UK). Fundoscopic examination revealed healthy optic discs. Tortuous and dilated retinal vessels in the right fundus, and to a far lesser extent on the left were noted, without exudation or hemorrhage present (Fig. 1A). The diagnosis of arteriovenous malformation was made, with no evidence of progression or visual consequence reported over seven years of annual evaluation (Fig. 1B). Visual acuity of the patient was 6/24 and 6/6 for the right and left eye respectively, having high hyperopia in the right eye and no refractive error in the left. Right eye vision had not improved significantly after attempted patching in early childhood. Visual acuity remained stable over time. The eyes were normally aligned, and extra-ocular movements were full. Visual fields to confrontation were normal. Intraocular pressure in both the right eye and left eye was 12 mmHg. Slit-lamp examinations of the anterior segments of both eyes were unremarkable.

At 14 years of age at routine follow-up, she complained of an intermittent right-sided headache and a pressure sensation around the left eye. At a follow up three years later, this gradually escalated to throbbing pain and discomfort behind both eyes. This pain was not related to ocular movement and did not wake the patient up at night. Retinal imaging was stable. Magnetic resonance imaging and angiography (MRI and MRA) was requested to exclude intracranial involvement (both at age 13 and 17 years). Neuroimaging was normal, ruling out central nervous system vascular abnormalities and ocular ultrasound had no observable malformations. Ophthalmic blood vessels on both eyes had no obvious vascular abnormalities, in contrast to the retinal vessels.

Color fundus photos and OCT imaging were in keeping with an AVM in the right eye; with retinal vessels in the right eye being both highly tortuous and dilated (Fig. 1A). Both eyes were imaged with swept-source OCTA (SS-OCTA, Plex Elite, Carl Zeiss Meditec AG). This demonstrated that the macrovessels were not passing through either the macula (Fig. 1C) or the foveal avascular zone (Fig. 1D). OCTA B-scan images of the right and left eye were analyzed in ImageJ to quantify retinal blood perfusion and then compared between eyes, in order to investigate the impact of this unilateral AVM (Fig. 1E). The eye with the AVM had significantly higher retinal blood perfusion than the unaffected eye. The percentage difference of blood flow between eyes reached a maximum of 1102% in the superior arcade (Fig. 1E 1 and 4). This trend continued with the percentage difference between the left and right inferior arcade being 836.2% (Fig. 1E 2 and 5), and 190.5% for the fovea (Fig. 1E 3 and 6).

## 3 Discussion

Retinal arteriovenous malformations (AVMs) are rare, congenital vascular abnormalities initially described in 1874 by Magnus.^2^ This condition is non-hereditary, and frequently arises unilaterally from the optic nerve extending into the periphery or the macula.^3^ High levels of retinal venous and arterial dilatation accompanied by a tortuous pattern are a key clinical sign, that was observed in the right eye of the patient. These malformations can often be seen in patients that are asymptomatic and vision typically remains stable.^4^ Retinal AVMs typically do not decrease visual acuity, however ocular complications may arise such as: vitreous hemorrhage, retinal artery occlusion, mechanical compression of the optic nerve, neovascular glaucoma and retinal vein occlusion.^3^

Congenital retinal macrovessels are the result of an atypical embryogenesis and believed to be formed during weeks 15–16 of gestation, and the underlying cause remains unknown.^4^ The condition was first described in 1869 by Mauthner as an “aberrant retinal vessel” and typically features a vein which crosses the central macula,^5^ however this was not seen in our patient. The presence of large aberrant veins in the right eye of our patient are better described as an AVM, rather than congenital retinal macrovessels.

OCTA is a fast form of non-invasive imaging of deep and superficial vascular layers in the retina. OCTA imaging in our case revealed that the retinal AVM can be classified as type 1, as the arteriolar capillary plexus was present between the communicating vessels. Intracranial AVMs do not commonly accompany type 1 malformations,^2^ and our case did not have any MRI findings on two occasions. A type 2 retinal AVM is defined as having arteriovenous communication without capillary or arteriolar segments intervening, and a Type 3 retinal AVM appears as large convoluted vessels.^2^

This is the third report of OCTA in a child with retinal AVM, but the first report of a type 1 AVM; and moreover the first report quantifying retinal blood flow associated with an AVM. The other two cases reported involved a 7-year-old female with a type 3 AVM, and a 6-year-old boy that had deteriorating vision with a type 3 AVM.^6^^,^^7^ In all cases, dilated vessels with high tortuosity were seen in the superficial layers of the retina on OCTA. The OCT B-scan of our patient also showed multiple abnormally large retinal vessels, which was also seen in 2 other children in 2015, however no shadowing artefact was present in the scan of our patient.^8^

This was the first case that was imaged with the widefield OCT Angiography Plex Elite. This machine allows visualisation of a wide field of blood vessels non-invasively, facilitating repeated imaging which is essential in monitoring change/stability over time. Amblyopia in children can occur by congenital retinal macrovessels crossing the fovea.^9^ Although the patient had anisometropic amblyopia, no vessels were crossing the fovea. No choroidal changes were identified, that could explain an axial length and unilateral high hyperopia and secondary amblyopia. This report presents the first case of AVM in an eye with both right anisometropic amblyopia and iris heterochromia. The patient has complete heterochromia having a brown iris on the right eye and a greenish brown iris on the left. The color of the iris is determined by the distribution and concentration of melanin, and is affected by physiologic and genetic factors.^10^ Although the condition is uncommon, most cases of heterochromia are benign.

## 4 Conclusions

AVMs usually remain stable, however regular follow-up is recommended to manage potential complications, if they arise.^11^ Analysis of blood flow in the eyes has shown that the AVM caused a profound vascular blood flow increase compared with the fellow eye. Neuroimaging should be considered to exclude intracranial involvement and rule out CNS vascular abnormalities. This report highlights the use of widefield OCTA as an important, non-invasive easily repeatable method of assessing and monitoring blood flow and differential flow between the affected and non-affected fellow eye.

## Patient consent

Written consent to publish this case has not been obtained. This report does not contain any personal identifying information.

## Funding

Supported by grants from the 10.13039/501100012618National Institute for Health Research Biomedical Research Centre at Moorfields Eye Hospital NHS Foundation Trust and UCL Institute of Ophthalmology, 10.13039/501100005302Onassis Foundation, 10.13039/501100004117Leventis Foundation, 10.13039/100010269The Wellcome Trust (099173/Z/12/Z), Moorfields Eye Charity, Retina UK, and the Foundation Fighting Blindness (10.13039/100011408USA).

## Authorship

All authors attest that they meet the current ICMJE criteria for Authorship.

## Financial disclosures

Ranjan Rajendram is a speaker for Carl Zeiss Meditec AG. No conflicting relationship exists for any other author (EW, MG, MM).

## Declaration of competing interest

Ranjan Rajendram is a speaker for Carl Zeiss Meditec AG. The following authors have no financial disclosures: EW, MG, MM.
